# Quaternized and Hyperbranched Amidoxime-Modified Ultra-High-Molecular-Weight Polyethylene Fiber for Uranium Extraction from Seawater

**DOI:** 10.3390/polym16233310

**Published:** 2024-11-27

**Authors:** Lijun Hu, Hongwei Han, Xuanzhi Mao, Xinxin Feng, Yulong He, Jiangtao Hu, Guozhong Wu

**Affiliations:** 1 Shanghai Institute of Applied Physics, Chinese Academy of Sciences, No. 2019 Jia-Luo Road, Jia-Ding District, Shanghai 201800, China; hulijun@sinap.ac.cn (L.H.);; 2 College of Science, Shanghai University, Shanghai 200444, China; hanhongwei@sinap.ac.cn; 3 Shaanxi Coal and Chemical Technology Research Institute Co., Ltd., Xi’an 710100, China

**Keywords:** uranium extraction from seawater, hyperbranched structure, anti-biofouling, UHMWPE fiber

## Abstract

The most promising material for uranium extraction from saltwater is generally acknowledged to be fibrous adsorbents. An irradiation-modified anti-biofouling ultra-high-molecular-weight polyethylene (UHMWPE-*g*-PGAO) fibrous adsorbent with a hyperbranched structure was synthesized. It exhibited adsorption capacities of 314.8 mg-U/g-Ads in aqueous solution and 4.04 mg-U/g-Ads in simulated seawater over a 28-day period. The ultra-high-molecular-weight polyethylene (UHMWPE) fiber was functionalized by covalently linking hyperbranched polyethyleneimine (h-PEI) to facilitate the migration of uranyl ions within the fibers. Additionally, amidoxime and quaternary ammonium groups were immobilized on the fiber surface to enhance uranium affinity and provide defense against marine organisms. This three-dimensional design of amidoxime and h-PEI-modified UHMWPE fiber retained more than 91.0% of its maximum adsorption capacity after undergoing five adsorption-desorption cycles. The UHMWPE-*g*-PGAO adsorbent exhibits significant antibacterial activity against *Escherichia coli* and *Staphylococcus aureus*, achieving an inactivation efficiency of over 99.9%. It is proved to be an innovative fiber adsorbent for uranium extraction from seawater for its biofouling resistance, robustness, and reusability.

## 1. Introduction

The ocean has been considered an important source of unconventional uranium [1]. To utilize this inexhaustible resource, a variety of fibrous adsorbents [2,3,4,5] with different adsorption groups, morphologies, and sizes has been developed [6], such as traditional organic polymer adsorption materials [7], novel metal-organic frameworks [8], covalent organic frameworks, and others [9,10]. Following the screening of adsorbents in marine field adsorption, amidoxime (AO) groups and their modified fiber adsorbents are considered the most effective for uranium adsorption [11,12]. However, long-term studies have also revealed innate deficiencies [1] of these AO-modified adsorbents. Ionic cross-linking occurred on the surface during adsorption due to electrostatic interactions between AO and uranyl ions, which restricted the further diffusion of uranyl ions inward, making it infeasible to utilize internal AO in the adsorbent [13]. Furthermore, marine biofouling not only compromises the mechanical integrity but also diminishes the adsorption efficiency and reusability of the adsorbent [14,15]. Expanding the adsorbent materials’ surface area can improve the adsorption capacity to a certain extent. However, the adsorption groups persist in a close packing state, which does not fully address the issue of diffusion obstruction. To eliminate obstacles to the diffusion of uranyl ions, a hyperbranched structure was first grafted onto the UHMWPE fiber to impede physical cross-linking, and quaternary ammonium groups were then grafted to the fiber to preclude biofouling [16,17]. A valuable material for marine field adsorption applications, the AO-based UHMWPE fiber created by pre-radiation grafting polymerization benefited from its structural design and showed high physical and chemical stability in seawater, rapid selective uranium adsorption, and sufficient adsorption capacity.

Due to their unique highly branched tree-like architectures, hyperbranched polymers (HBPs) have attracted considerable interest [18,19], a large cavity, special physical/chemical properties, and versatile functionalities. In various HBPs, hyperbranched h-PEI is a hydrophilic macromolecule containing numerous amine groups; this structure has been modified with different functional groups to enhance the adsorbent’s ability to capture and selectively bind heavy metals, including U (VI) [20], Cr (VI), and Cu (II) [21].

Herein, a uranium adsorbent containing antibacterial groups and a three-dimensional dendritic structure was prepared via pre-radiation grafting polymerization and subsequent chemical modification. There are three major breakthroughs in this study as compared to previous research. First, the packing density of adsorbent groups is reduced by grafting high-free-volume h-PEI to mitigate the impact of ionic crosslinking, which commonly affects uranyl ion diffusion during uranium adsorption. Second, the bifunctional AO and h-PEI network strengthens the affinity of the AO ligand through a synergistic effect between the two, offering abundant functional groups that improve uranyl adsorption capacity. Moreover, it provides greater hydrophilicity than single poly (amidoxime). Finally, this adsorbent exhibits exceptional antimicrobial activity against marine bacteria at high concentrations, in addition to its enhanced adsorption capacity.

## 2. Materials and Methods

### 2.1. Materials

The reagents and characterization techniques used in this experiment are listed in the Supplementary File.

### 2.2. Preparation of UHMWPE Fibrous Adsorbent

#### 2.2.1. Pre-Radiation Grafting Polymerization

**Scheme 1:**
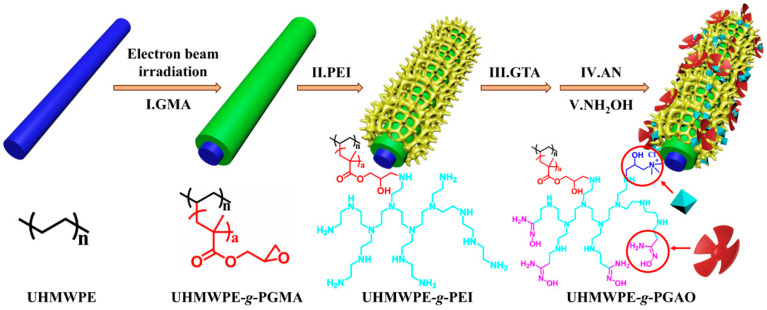
Preparation process of UHMWPE fibrous adsorption materials with hyperbranched structure.

Scheme 1 illustrates the process of fabricating the adsorbent material from UHMWPE fibers. Initially, the fibers were irradiated at 100 kGy using an electron beam accelerator and then stored in a refrigerator at −15 °C for subsequent experiments. For this, 0.5 g of Tween 20 was dissolved in a mixed solution of 80 mL of DI (deionized water) and 15 mL of GMA, stirring continuously with a magnetic stirrer for 4 h. The mixed solution was transferred into a 150 mL conical flask, and then 1 g of UHMWPE fiber was incorporated. Further, nitrogen was introduced into the flask for 20 min, and then the conical flask was placed in a water bath to react at 65 °C for 4 h. Upon completion of the reaction, the sample was removed, thoroughly cleaned three times with ethanol and deionized water, and baked at 60 °C until the weight reached a constant. The grafted fiber was named UHMWPE-*g*-PGMA, and the grafting rate D_g_ was calculated using Equation (1) [22]:

D*g* = (W_1_ − W_0_)/W_0_ × 100% (1)

In the formula, W_0_ and W_1_ represent the fiber mass before and after graft polymerization, respectively. The graft degree of PGMA on UHMWPE fiber was 129%.

#### 2.2.2. Introduction of Poly-Amino Hyperbranched Structures and Quaternary Antimicrobial Groups

For this, 25 g of PEI was added into 75 mL of 1,4-dioxane solution and stirred continuously with a magnetic stirrer for 1 h until PEI was completely dissolved and the solution was clear and transparent. Subsequently, 0.5 g UHMWPE-*g*-PGMA fiber was added into the above solution, and the temperature was maintained at 80 °C for 4 h while ensuring an inert nitrogen atmosphere. Following the reaction, the fibers were removed, rinsed with ethanol and deionized water for 3 times, and then baked in the oven at 60 °C for 6 h. The fiber with hyperbranched structure is named UHMWPE-*g*-PEI.

UHMWPE-*g*-PEI fiber was put into an aqueous solution containing 10 wt% 2,3-epoxypropyl trimethyl ammonium chloride (GTA) and reacted at 80 °C for 6 h, and then quaternary ammonium salt was introduced. The product was coded as UHMWPE-*g*-GTA.

#### 2.2.3. Introduction of Adsorption Groups

The UHMWPE-*g*-GTA fiber was placed in a solution of *N*,*N*-dimethylformamide (DMF) containing 20% acrylonitrile, and then nitrogen was bubbled for 20 min to remove oxygen in the solution. The reaction took place at a temperature of 45 °C for a duration of 4 h. Following, the fibers were thoroughly washed using ethanol and deionized water and dried in the oven at 60 °C for 6 h. The obtained sample was referred to as UHMWPE-*g*-PGAN.

Dissolve hydroxylamine hydrochloride (5.0 g) in deionized water (50 mL), then use sodium hydroxide to adjust the pH of the resulting solution to approximately 7.0, then add 50 mL dimethyl sulfoxide (DMSO), and stir the above mixed solution in a magnetic stirrer for 10 min. UHMWPE-*g*-PGAN fiber was placed in the above mixture and reacted at 70 °C for 3 h. The sample was removed and subsequently rinsed with ethanol, followed by drying in a vacuum oven set at 60 °C. The fibrous adsorbent material named UHMWPE-*g*-PGAO was prepared. Similarly, adsorption groups were introduced into UHMWPE-*g*-PEI, resulting in the adsorbent referred to as UHMWPE-*g*-PGPEAO, which lacks the quaternary ammonium salt structure. The content of AO (C_AO_) on the fiber was determined using Equation (2) [23]:

C_AO_ = (m_AO_ − m_AN_)/(33 × m_AO_) × 1000 mmol·g^−1^ (2)

where m_AN_ and m_AO_ denote the mass of grafted UHMWPE fiber prior to undergoing the amidoximation reaction and following its completion, respectively. In this study, C_AO_ of the UHMWPE-*g*-PGAO fiber is 3.3 mmol·g^−1^.

### 2.3. Uranium Adsorption Experiments

#### 2.3.1. Uranium Adsorption in Aqueous Solution

Six 100 mL bottles containing 8 ppm uranium solutions were prepared, with the pH of each solution adjusted individually from 4.0 to 9.0. Subsequently, approximately 10 mg of UHMWPE-*g*-PGAO fiber was added to each uranium solution with varying pH levels. The adsorption trials were performed at a controlled temperature of 25 °C with a stirring speed set at 120 rpm within a temperature-regulated oscillator. The concentrations of residual U(VI) were quantified using ICP-MS analysis. Finally, the capacity of the adsorbents was determined based on Equation (3):

Q_e_ = V/m × (C_0_ − C_e_) (3)

where Q_e_ indicates the capacity for adsorption and V stands for the solution volume. The mass of adsorbents is indicated by m, C_0_ is the concentration before adsorption, and C_e_ reflects the concentration following the adsorption process. Samples were collected after 24 and 48 h for further analysis.

After determining the optimal pH value for uranium adsorption on UHMWPE-*g*-PGAO fiber, further investigation was conducted to investigate the adsorption kinetics of uranium by UHMWPE-*g*-PGAO fiber. A 100 mL, 8 ppm uranium solution was prepared, and the optimal pH condition (pH = 5.0) was selected for the subsequent adsorption test. An amount of fiber adsorbent (10 mg) was added to the previously discussed solution. Samples were collected at different time intervals to measure the remaining concentration of uranium in the solution. Finally, the isothermal adsorption experiment is explored by changing the initial concentration of uranium (10–60 ppm, respectively), repeating the above experimental steps, and waiting for the adsorption to reach equilibrium, thus calculating the maximum adsorption capacity of the fiber.

#### 2.3.2. Batch Uranium Adsorption in Simulated Seawater

For the adsorption experiments, natural seawater was collected from the coastal region of Zhoushan in Zhejiang Province, China, located at coordinates 122.20° E and 30.00° N. After filtering out the sediment with a microporous membrane, the seawater was used for subsequent experiments. To promptly assess the effectiveness of the adsorbents in seawater, uranium was intentionally added to the seawater. To be specific, a specific quantity of standard solutions containing uranium and vanadium was introduced into the seawater to ensure the concentration of uranium and vanadium is 330 ppb and 200 ppb, respectively. The rise in vanadium ion concentration is attributed to its significant impact on the process of uranium adsorption in seawater. The levels of various metal ions, such as Fe, Co, Ni, Cu, Zn, Pb, Mg, and Ca, were maintained at values representative of actual seawater conditions. About 10 mg of UHMWPE-*g*-PGAO fiber was added to the above solution, and samples were periodically collected and subjected to microwave digestion. The solution obtained by digestion was diluted to measure and evaluate the adsorption performance of the adsorbed fiber by ICP-MS.

### 2.4. Antibacterial Property

For the evaluation of antibacterial effectiveness, the Gram-negative strain *E. coli* ATCC 8739 and the Gram-positive strain *S. aureus* ATCC 6538 were chosen as the target organisms of the UHMWPE-*g*-PGAO fiber following the dilution plate counting method outlined in Chinese standard GB/T 20944 [24,25,26]. Specifically, a aseptic conical flask of phosphate-buffered saline (PBS, pH 7.3, 70 mL) at a concentration of 30 mM/L was supplemented with 0.75 g fiber. The bacterial strains (1 × 10^5^ CFU/mL) were introduced into the mixture at a concentration of 5 mL and incubated at 150 rpm for 18 h at 24 °C in a temperature-controlled incubator. A volume of 1 mL of the supernatant underwent a tenfold dilution before being spread onto agar plates. Following an incubation time of 24 h (37 °C) in the incubator, colony counting was conducted. The inhibition rate (R_1_) was determined using Equation (4).

R_1_ = (C_0_ − C_S_)/C_o_ × 100% (4)

C_0_ and C_S_ denote the colony counts for the test and control samples, respectively. Here, the UHMWPE-*g*-PGPEAO fiber was utilized as the standard control sample.

## 3. Results and Discussion

### 3.1. Characterization of the UHMWPE-g-PGAO Fiber

#### 3.1.1. Chemical Structure and Morphology

**Figure 1:**
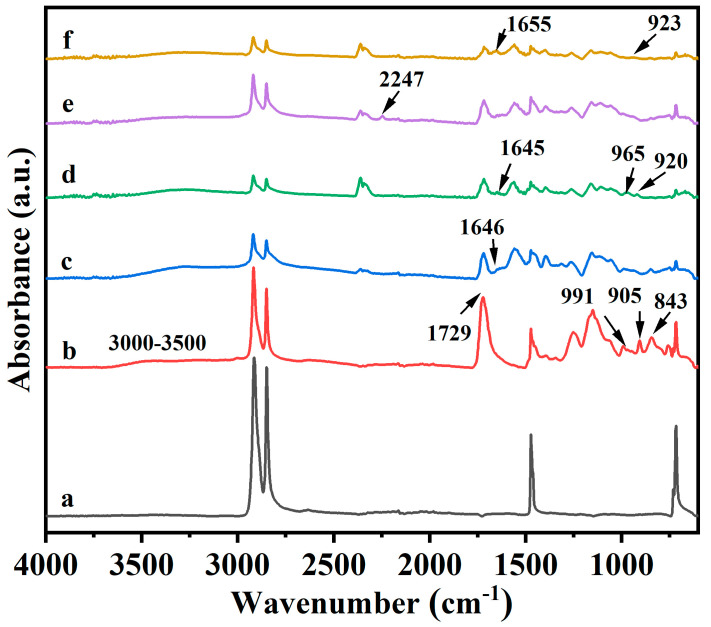
FTIR spectra of the UHMWPE (a), UHMWPE-*g*-PGMA (b), UHMWPE-*g*-PEI (c), UHMWPE-*g*-GTA (d), UHMWPE-*g*-PGAN (e), and UHMWPE-*g*-PGAO (f) fibers.

Figure 1 displays the FTIR spectra for both UHMWPE and UHMWPE-*g*-PGAO fibers, wherein the corresponding signals are carefully assigned. For the spectrum of pristine UHMWPE fiber, the peaks at 2906, 2846, 1468, and 715 cm^−1^ are regarded as indicative of the stretching and bending vibration of methylene [22], respectively. After graft polymerization, the grafted UHMWPE fiber demonstrates a peak around 1729 cm^−1^, ascribing to C=O stretching vibration, the ester groups of grafting chains. Additionally, there are peaks identified at 991, 905, and 843 cm^−1^, which are consistent with the vibrations of the epoxy group [27]. The above analysis confirmed that PGMA has been successfully covalently grafted onto the UHMWPE fiber surface. After PEI reacts with the epoxy groups, the epoxy groups disappear, and O-H and N-H groups appear. The peaks belonging to the epoxide group disappeared in the spectra of UHMWPE-*g*-PGMA fiber, and peaks corresponding to O-H and N-H vibrations appeared at 3000–3500 cm^−1^; in addition, a new peak at 1646 cm^−1^ is also attributed to N-H. The above analyses illustrate that hyperbranched PEI chains were successfully introduced into UHMWPE-*g*-PGMA fiber. For UHMWPE-*g*-GTA fiber, three absorption peaks centered at 1645, 965, and 920 cm^−1^ corresponding to quaternary ammonium salt were observed [28]. The occurrence of a stretching vibration peak of CN at 2247 cm^−1^ illustrates that acrylonitrile has been successfully attached onto the fiber surface through the Michael addition reaction [29]. The elimination of this peak suggests that nitrile groups have been transformed into AO groups following the reaction with NH_2_OH. After amidoximation reaction, significant absorption bands indicative of NH, OH, C=N, and N-O groups were detected at wavenumbers of 3000–3500, 1655, and 923 cm^−1^, respectively [23]. The results confirmed that AO adsorption groups and antibacterial groups were introduced onto UHMWPE fibers simultaneously.

**Figure 2:**
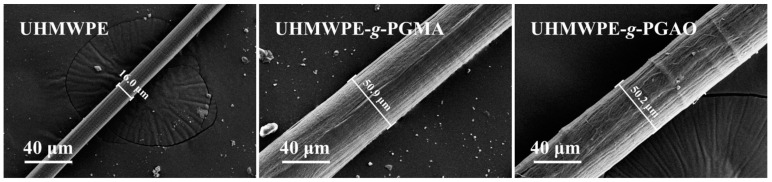
SEM images of UHMWPE, UHMWPE-*g*-PGMA, and UHMWPE-*g*-PGAO fibers.

SEM images (Figure 2) of pristine UHMWPE fiber and the modified fibers were provided to compare their morphologies and investigate potential damage that may have occurred during the modification process. The raw fiber exhibits a smooth, flat surface with visibly native striations. After PGMA grafting, longitudinal stripes appear on the fiber surface, leading to an increase in fiber diameter. Subsequent chemical reactions cause the fiber to exhibit regular bulges due to the swelling of the grafted layer. Importantly, no cracks were observed on the UHMWPE-*g*-PGAO fiber surface, suggesting that the original UHMWPE fiber experienced minimal adverse impact.

#### 3.1.2. Thermal Property

**Figure 3:**
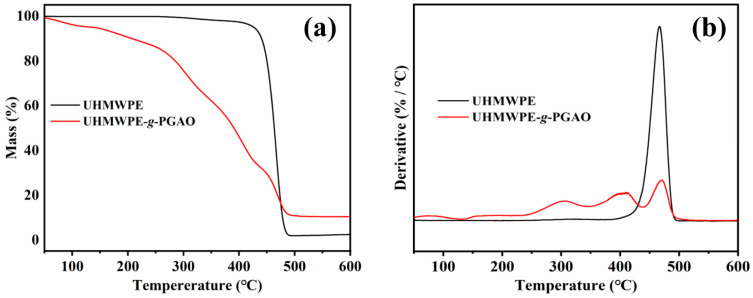
TG (**a**) and DTG (**b**) curves of UHMWPE and UHMWPE-*g*-PGAO fibers.

TG testing analysis was performed, with the findings illustrated in Figure 3. The thermogram for UHMWPE fiber displays a distinct, single-step degradation process. It reveals an initial decomposition temperature of 447 °C and reaches a peak decomposition temperature of 467 °C [30], leaving no residue at 600 °C. However, the four-step thermal decomposition behavior is clearly observed in the TG curves of modified fibers with different chemical structures. The initial quality loss corresponds to the evaporation of water from the surface of the material, and the subsequent two steps of thermal decomposition result from the degradation of graft chains and functional groups due to the low molecular weight and poor thermal stability. The degradation at 467 °C conformed to the diagram of pristine UHMWPE fiber [31]. The lower thermal stability is due to the crystallinity associated with the grafted chains, as well as the presence of the functional groups that have been introduced. Nevertheless, it is considered that these differences have no effect on its application in uranium collection from seawater because the normal operating temperatures of adsorption, elution, and regeneration processes do not exceed 40 °C.

### 3.2. Uranium Adsorption

#### 3.2.1. Impact of pH Levels on the Uranium Adsorption

**Figure 4:**
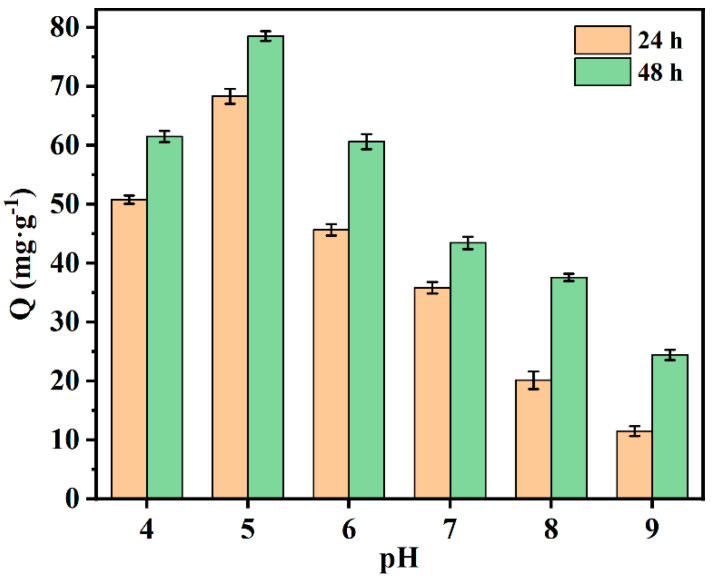
pH effects on U(VI) adsorption of UHMWPE-*g*-PGAO fiber in aqueous solutions (C_0_ = 8 ppm, V = 100 mL, m_Ads_ = 10 mg, and T = 25 °C).

pH has a significant influence on uranium adsorption by affecting the distribution of uranyl ion species as well as altering the surface charge characteristics of the adsorbent. As illustrated in Figure 4, the adsorption amounts rise within the pH range of 4.0–5.0, peak at pH 5.0, and subsequently decline with further pH increments from 6.0 to 9.0. At pH < 5.0, high concentrations of H^+^ ions protonate the adsorption groups, so the repulsive electrostatic interaction occurring between adsorbents with a positive charge and cationic forms of uranium leads to low adsorption capacity under acidic conditions. The binding site is gradually deprotonated with increasing pH, and then the R-NO anion. The single pair of electrons on the amino nitrogen interacts with the uranyl cation via electrostatic forces and complexation, resulting in the formation of stable chelates. This process effectively enhances the adsorption capacity for uranium (U-VI). In an alkaline environment, anionic uranium species increase with the increase in pH level [6,32]. As the pH level rises, the prevalence of anionic uranium species also increases, ultimately becoming the predominant form of uranium. This shift negatively impacts the efficiency of adsorption processes. Thus, the optimal initial pH is 5.0, and subsequent experiments are also conducted at this condition. Figure 4 illustrates that the adsorption capacity of UHMWPE-*g*-PGAO achieved 85.4% (68.31 mg-U/g-Ads) after 24 h and increased to 98.1% (78.51 mg-U/g-Ads) following 48 h. The increase in adsorption capacity with time is due to the fact that the adsorption has not reached equilibrium within 48 h, prior to which the adsorption capacity is positively correlated with time. This result demonstrated that UHMWPE-*g*-PGAO could be an effective material for the extraction of U(VI) from seawater.

#### 3.2.2. Adsorption Kinetics

**Figure 5:**
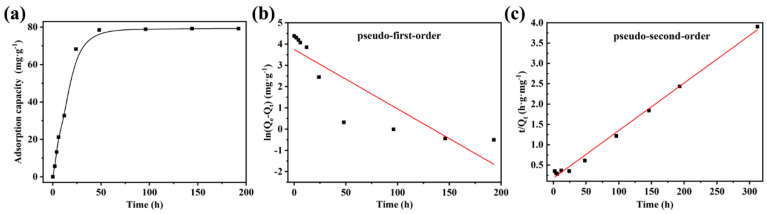
(**a**) The dependence of adsorption capacity on time; (**b**) Model-1 and (**c**) Model-2 (pH = 5.0, C_0_ = 8 ppm, V = 100 mL, m_Ads_ = 10 mg, and T = 25 °C).

Adsorption kinetics is an important way to fully understand the adsorption process. As shown in Figure 5a, the UHMWPE-*g*-PGAO fiber presents a rapid adsorption response to uranyl ions. Figure 5b,c illustrate the pseudo-first-order (Model-1) and pseudo-second-order adsorption models (Model-2), which are expressed in Equations (5) and (6), respectively.

ln (Q_e_ − Q_t_) = ln (Q_e_) − k_1_ t (5)

t/Q_t_ = 1/(k_2_ × Q_e_^2^) + t/Q_e_ (6)

Here, Q_e_ and Q_t_ refer to the quantity of U(VI) adsorbed at equilibrium time and any specific time, respectively, and k_1_ and k_2_ denote the kinetic rate constants. The correlation coefficient of Model-2 is 0.9937, which is higher than 0.7659 of Model-1 (Table 1). Furthermore, the computed value for adsorption capacity closely aligns with the results derived from experimental observations. The results suggested that the adsorption of U(VI) onto UHMWPE-*g*-PGAO aligns with the Model-2; this is in accordance with the chemisorption mechanism as a rate control step. It is highly probable to infer that the adsorption behavior may be related to the valence force between the adsorbent and uranyl ion.

#### 3.2.3. Isothermal Adsorption

**Figure 6:**
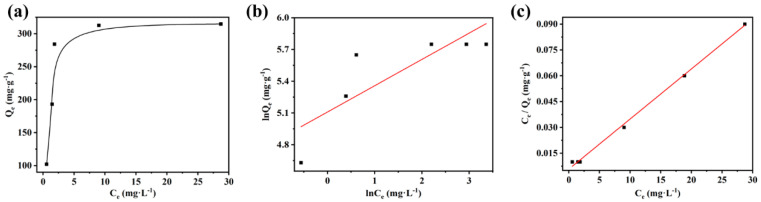
(**a**) Adsorption isotherm; linear fitting by (**b**) Freundlich and (**c**) Langmuir isothermal adsorption models. (pH = 5.0, V = 100 mL, m_Ads_ = 10 mg, and T = 25 °C).

The effect of initial uranium concentration on adsorption behavior of the UHMWPE-*g*-PGAO adsorbent was investigated over a range of 10 to 60 mg/L at 25 °C for 5 d. The adsorption isotherm (Figure 6a) represents the equilibrium distribution of adsorbates between the aqueous and the adsorbent. The adsorption data were modeled by the Langmuir and Freundlich isothermal models to evaluate the maximum adsorption capacity of UHMWPE-*g*-PGAO for uranium, as depicted in Equations (7) and (8), respectively.

C_e_/Q_e_ = 1/(K_L_ × Q_m_) + C_e_/Q_m_ (7)

ln(Q_e_) = ln(K_F_) + 1/n × ln(C_e_) (8)

K_L_ is the Langmuir adsorption constant, representing the affinity of the adsorbent to uranium; Q_e_, C_e_, and Q_m_ are the adsorption capacities of UHMWPE-*g*-PGAO, the uranium concentration in the aqueous phase, and the maximum adsorption capacity of UHMWPE-*g*-PGAO, respectively. K_F_ is the Freundlich adsorption constant, while 1/n represents the adsorption intensity. The linearized plots of the two models are given in Figure 6b,c. The fitting parameters are displayed in Table 2. The R^2^ of the Langmuir model (0.9983) is bigger than that of the Freundlich model (0.4030), illustrating that the adsorption behavior of UHMWPE-*g*-PGAO is uniform chemical adsorption. In addition, Q_m_ (327.9 mg/g) derived from the Langmuir model aligns closely with the experimental Q_e_ (314.8 mg/g). Both the Q_m_ and Q_e_ significantly surpass those of fibrous AO-modified adsorbents from our previous studies and other literature [23,33]. This can be attributed to hyperbranched PEI inhibiting ionic cross-linking on the fiber surface during adsorption, thereby allowing uranyl ions to penetrate deeper into the fiber structure.

#### 3.2.4. Uranium Adsorption Tests in Simulated Seawater

**Figure 7:**
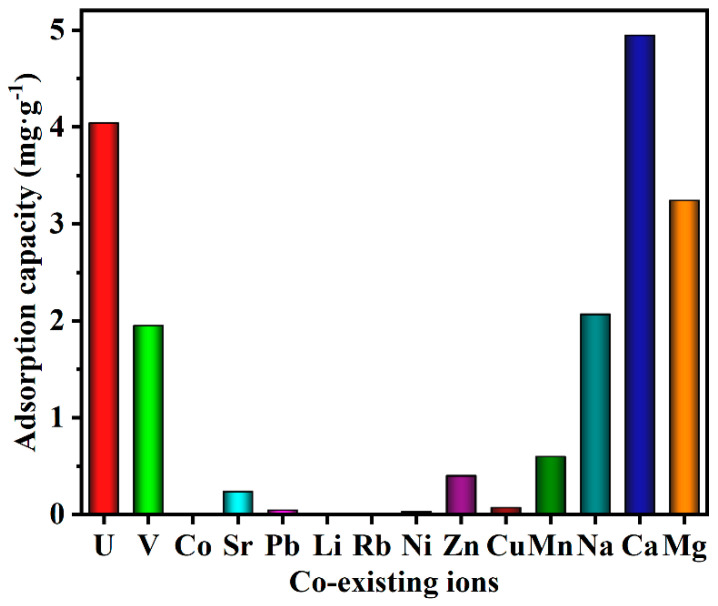
The adsorption capacities of UHMWPE-*g*-PGAO fiber for various ions in simulated seawater after 28 d of adsorption (V = 400 mL, m_Ads_ = 10 mg, and T = 25 °C).

Selectivity of UHMWPE-*g*-PGAO for ions was evaluated in simulated seawater. Compared with the fibrous AO-modified UHMWPE in our previous study, hyperbranched PEI and quaternary ammonium groups facilitate adsorption capacity enhancement in this study. It was proved that the UHMWPE-*g*-PGAO exhibited a high adsorption capacity and a good selectivity for UO_2_^2+^ (Figure 7): after 28 d of adsorption, the uranium adsorption capability was 4.04 mg/g, which significantly exceeded that of the other coexisting ions except Ca^2+^, although the concentrations of the other coexisting ions were much higher than that of uranium. This can be attributed to the planarity, and the presence of electronegative atoms in a suitable arrangement of AO allows it to form stable complexes with U and V ions [12], which is not as effective with other ions. In addition, it was reported in relevant literature that the binding energy of AO with U is higher than that of AO with V [34], thus the adsorption capacity of U was higher than that of V, and the value of U/V is larger than 2, revealing the excellent selectivity of UHMWPE-*g*-PGAO for uranium. The absorption of calcium (4.95 mg/g) was higher than that of uranium, mainly attributed to the concentration of Ca^2+^ (411 ppm) in seawater, which is much higher than that of UO_2_^2+^.

#### 3.2.5. Regeneration Performance

**Figure 8:**
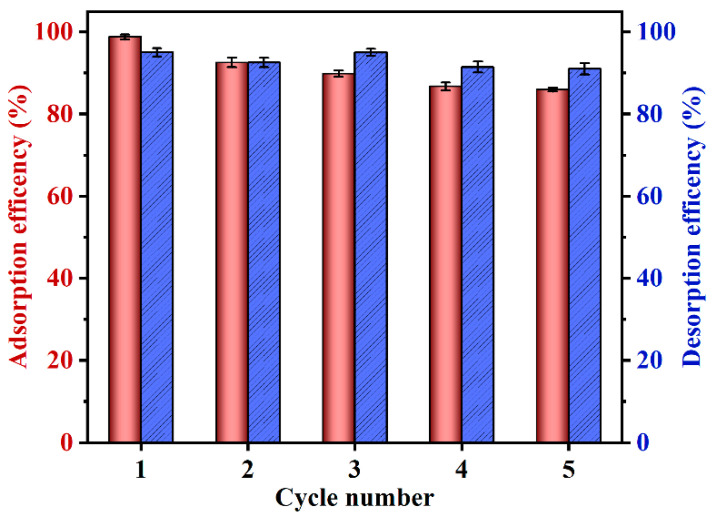
Varieties of uranium removal rates during the 5 adsorption-desorption cycles of UHMWPE-*g*-PGAO fiber in an aqueous solution of uranium (C = 8 ppm, pH = 5.0, m_Ads_ = 10 mg, and T = 25 °C).

The reusability of UHMWPE-*g*-PGAO is critical for industrial applications, as reusability affects the economic cost of the adsorbent, which was assessed by 5 adsorption-desorption tests. After the tests, UHMWPE-*g*-PGAO maintained an adsorption capacity for uranium exceeding 85.9% of the initial value while achieving an elution efficiency of 91.0% (Figure 8), illustrating UHMWPE-*g*-PGAO can be repeatedly used to extract uranium from seawater. UHMWPE-*g*-PGAO is amazingly robust, resulting in excellent reusability, which has very little loss during extraction, desorption, and rinsing.

#### 3.2.6. Antimicrobial Activity

**Figure 9:**
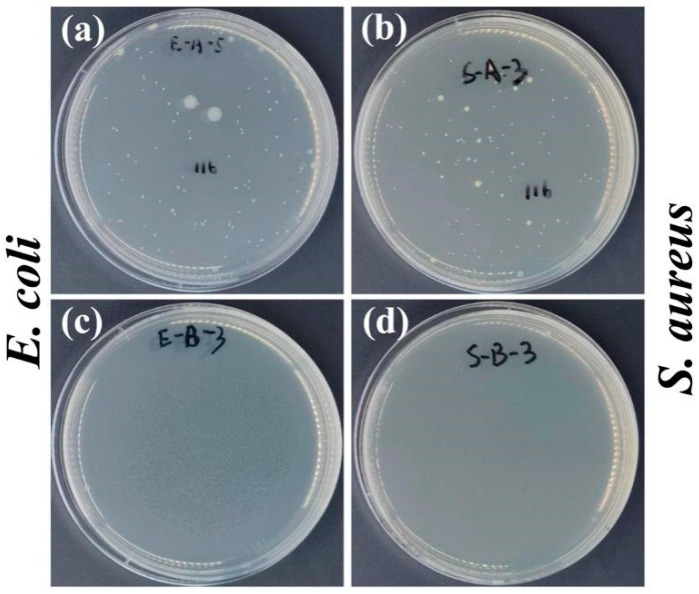
Antibacterial activity of UHMWPE-*g*-PGPEAO adsorbent against *E. coli* (**a**) and *S. aureus* (**b**); antibacterial activity of UHMWPE-*g*-PGAO against *E. coli* (**c**) and *S. aureus* (**d**).

**Figure 10:**
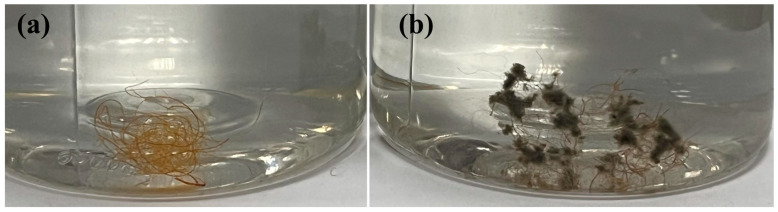
The pictures of UHMWPE-*g*-PGAO (**a**) and UHMWPE-*g*-PGPEAO (**b**) deployed in nature seawater for 28 d.

When extracting uranium from seawater, the antibacterial activity of the adsorbent is crucial, as the proliferation of marine microorganisms on the material’s surface can significantly diminish its adsorption performance [35]. Figure 9 clearly demonstrates the highly effective antibacterial activity of the UHMWPE-*g*-PGAO adsorbent. The UHMWPE-*g*-PGPEAO lacked resistance to *E. coli* and *S. aureus.* However, after the structural modification with quaternary ammonium salt, the adsorbent obtained strong antibacterial properties, and the inactivation efficiency was more than 99.9%. To further explore its marine applicability, UHMWPE-*g*-PGAO and UHMWPE-*g*-PGPEAO fibers were placed in real seawater without any treatment for adsorption. As shown in Figure 10a, no microbial attachment was observed on the UHMWPE-*g*-PGAO adsorbent, which exhibited a darker color due to uranium adsorption. In contrast, the surface of the adsorbent without grafting GTA is covered with black microorganisms (Figure 10b), which isolates the uranyl ions and the amidoxime groups, thus inhibiting the interaction between AO and UO2^2+^ and invalidating the adsorbent. The exceptional antibiofouling characteristics of UHMWPE-*g*-PGAO are instrumental in showcasing its substantial adsorption capacity and impressive recyclability for uranium extraction from seawater. The antimicrobial activity of the UHMWPE-*g*-PGAO adsorbent probably arises from the following factors. First, bacterial cell membranes with negative charge are attracted by ammonium groups and attach to the adsorbent. Second, the hydrophobic groups penetrate the bacterial cell wall and block the hazardous metabolites in the cell, denaturing and inactivating the cell membrane proteins. Finally, the alkyl hydrophobic chains pierce the cell wall and attach to the cell membrane, altering the cell membrane permeability. RNA, DNA, ions, and other components of the cell begin to escape, eventually killing the bacteria [36].

#### 3.2.7. Analysis of Adsorption Mechanism

**Figure 11:**
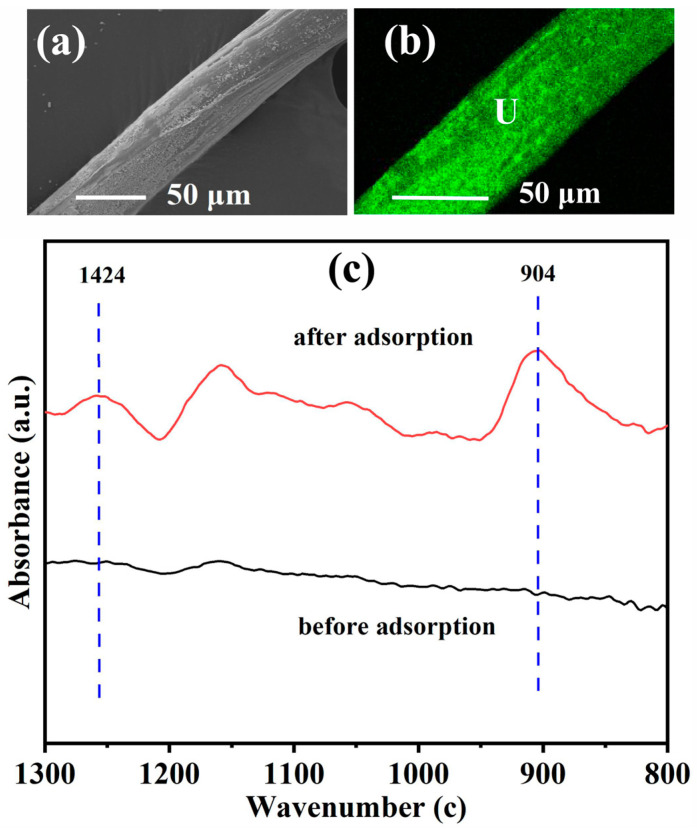
SEM image (**a**), EDS energy distribution (**b**), and ATR-FTIR (**c**) spectra of UHMWPE-*g*-PGAO after uranium collection.

SEM and energy dispersion spectroscopy (EDS) analysis showed that uranium was uniformly distributed on the UHMWPE-*g*-PGAO adsorbent, which confirmed the successful adsorption of uranium by the adsorbent (Figure 11a,b). UHMWPE-*g*-PGAO adsorbent after adsorption was abbreviated as UMWPE-*g*-PGAO-U. Moreover, in the FTIR spectra of UHMWPE-*g*-PGAO after adsorption (Figure 11c), a new characteristic peak of UO_2_^2+^ appeared at 1424 and 904 cm^−1^ [19,37], demonstrating that uranium has been successfully adsorbed onto the surface of the UHMWPE-*g*-PGAO adsorbent.

**Figure 12:**
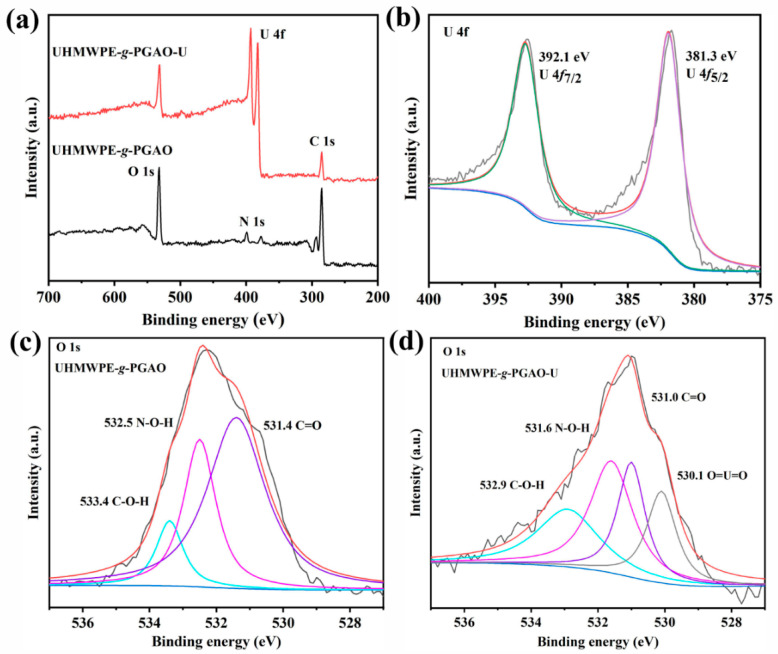
The wide-scan XPS spectrum of UMWPE-*g*-PGAO-U and UMWPE-*g*-PGAO fibers (**a**); high-resolution XPS spectra of U 4f (**b**), O 1s for UHMWPE-*g*-PGAO (**c**), and UMWPE-*g*-PGAO-U (**d**) fibers.

**Figure 13:**
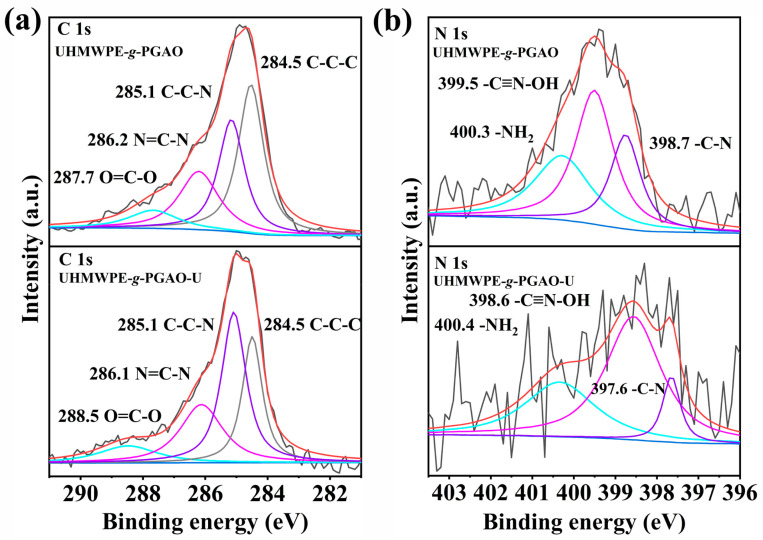
(**a**) C 1s and (**b**) N 1s XPS spectrum of UHMWPE-*g*-PGAO and UMWPE-*g*-PGAO-U before and after uranium adsorption.

For detailed analysis of the chemical composition of UHMWPE-*g*-PGAO-U, XPS was performed. Compared with the XPS spectrum of UHMWPE-*g*-PGAO, two new peaks were found at 381.3 eV and 392.1 eV, attributed to U 4f7/2 and U 4f5/2, respectively (Figure 12a,b). The high-resolution N 1s spectrum shows the peaks at 399.5 and 398.7 eV shift to 398.6 and 397.6 eV, belonging to C=N-OH and C-N, respectively (Figure 13). Additionally, the high-resolution spectra of O 1s of UHMWPE-*g*-PGAO and UHMWPE-*g*-PGAO-U (Figure 12c,d) show shifts in the peaks associated with C-O-H, N-O-H, and C=O, from 533.4, 532.5, and 531.4 to 532.9, 531.6, and 531.0 eV, respectively, indicating that uranyl is predominantly chelated to the oxime group [38]. Following the adsorption of uranium, an additional peak belonging to O=U=O emerges, indicating successful adsorption of uranium.

## 4. Conclusions

A uranium extraction material based on UHMWPE with a hyperbranched structure, named UHMWPE-*g*-PGAO, was successfully synthesized by attaching AO and quaternary ammonium groups to UHMWPE fibers. The UHMWPE-*g*-PGAO fiber exhibited a notable adsorption capacity of 314.8 mg-U/g-Ads in aqueous solution and 4.04 mg-U/g-Ads in simulated seawater over a 28-day period. In the antimicrobial test, UHMWPE-*g*-PGAO absorbent killed 99.9% of *E. coli* as well as *S. aureus*, exhibiting remarkable bacteria and biofouling resistance. Furthermore, following five adsorption-desorption cycles, the adsorbent retained over 85.9% of its adsorption capacity and more than 91.0% of desorption efficiency, indicating its favorable reusability and renewability. This study introduces novel insights into the synthesis of uranium extraction materials and promotes the practical application of the adsorbent for extracting uranium from seawater.

## Figures and Tables

**Table 1:** Fitting parameters of Model-1 and Model-2.

Model-1			Model-2		
k_1_ (h^−1^)	Q_e_ (mg/g)	R^2^	k_2_ (g·mg^−1^·h^−1^)	Q_e_ (mg/g)	R^2^
2.8 × 10^−2^	42.44	0.7659	7.6 × 10^−4^	85.47	0.9944

**Table 2:** Fitting parameters of Freundlich and Langmuir isothermal adsorption models.

Freundlich Model			Langmuir Model		
K_F_	1/n	R^2^	Q_m_ (mg/g)	K_L_ (L/mg)	R^2^
8.9 × 10^−6^	2.4473	0.4030	327.9	1.3737	0.9983

## Data Availability

The original contributions presented in the study are included in the article/Supplementary Material, further inquiries can be directed to the corresponding authors.

## Supplementary Materials

The following supporting information can be downloaded at: https://www.mdpi.com/article/10.3390/polym16233310/s1.
