# Supplementary material for: Quaternized and Hyperbranched Amidoxime-Modified Ultra-High-Molecular-Weight Polyethylene Fiber for Uranium Extraction from Seawater
Source: Polymers (Basel). 2024 Nov 27;16(23):3310. doi: 10.3390/polym16233310 (PMC11644467; doi:10.3390/polym16233310)
Supplement: Supplementary file 1 [file polymers-16-03310-s001.zip › polymers-3291854-supplementary.pdf]

Supplementary File

Quaternized and hyperbranched amidoxime-modified UHMWPE fiber for uranium extraction from seawater

Li-jun Hu[&1], Hong-wei Han[&2], Xuan-zhi Mao[1], Xin-xin Feng[1], Yu-long He[3], Jiang-tao Hu*[1], Guo-zhong Wu*[1]

1. Shanghai Institute of Applied Physics, Chinese Academy of Sciences, No. 2019 Jia-Luo Road, Jia-Ding District, Shanghai, 201800, China

2. College of Science, Shanghai University, Shanghai 200444, People's Republic of China

3. Shaanxi Coal and Chemical Technology Research Institute Co., Ltd., shaanxi, 710100, China

[&] These authors contributed equally to this paper and should be considered co-first authors

* Corresponding authors: Guo-zhong Wu (Email address: wuguozhong@sinap.ac.cn); Jiang-tao Hu (Email address: hujiangtao@sinap.ac.cn)

S1. Materials

UHMWPE fiber was obtained from Beijing Tongyizhong Special Fiber Technology Development Co., Ltd., with a density of 0.97 g/cm^3 and an average molecular weight of approximately 3 million. Glycerol methacrylate (GMA), acetone, ethanol, methanol, acrylic acid (AR), sulfuric acid (AR), hydrochloric acid (AR), nitric acid (AR), 1,4-dioxane, 2,3-epoxypropyltrimethylammonium chloride (GTA), acrylonitrile (AN), N,N-dimethylformamide (DMF), dimethyl sulfoxide (DMSO), hydroxylamine hydrochloride ($NH_2OH·HCl$), sodium hydroxide (AR), and potassium hydroxide (AR) were provided by Sinopharm Chemical Reagent Co., Ltd. Uranium standard solution (1000 ppm), polyethyleneimine (PEI; M.W. 600), and Tween 20 were provided by Shanghai Macklin Biochemistry Co., Ltd. The aforementioned reagents were used as received without further purification.

S2. Characterization Techniques

Attenuated total reflection Fourier-transform infrared (ATR-FTIR) spectra were acquired in the range of 600 to 4000 cm^{-1} using a Bruker Tensor 207 infrared spectrometer (Germany), with an accumulation of 32 scans and a wavenumber resolution of 4 cm^{-1}.

Thermogravimetric (TG) analyses were conducted using a TG 209 F3 Tarsus (NETZSCH, Germany) instrument over a temperature range of 25 to 600 ℃ at a heating rate of 10 ℃·min^{-1}, with a nitrogen flow rate of 10 mL·min^{-1}. The initial degradation temperature (Tdi) is defined as the temperature at which the sample weight loss reaches 5 wt%.

The surface morphological changes of both untreated and treated cotton fabrics were examined using an FEI Quanta-250 (USA) scanning electron microscope.

The chemical composition of the samples was analyzed using a Kratos Axis-Ultra DLD XPS instrument (Japan), and the data were processed using XPSPEAK41 software.
